# Evaluation of particle radiotherapy for the re-irradiation of recurrent intracranial meningioma

**DOI:** 10.1186/s13014-018-1026-x

**Published:** 2018-05-08

**Authors:** Rami A. El Shafie, Maja Czech, Kerstin A. Kessel, Daniel Habermehl, Dorothea Weber, Stefan Rieken, Nina Bougatf, Oliver Jäkel, Jürgen Debus, Stephanie E. Combs

**Affiliations:** 1 Department of Radiation Oncology, University Hospital of Heidelberg, Im Neuenheimer Feld 400, 69120 Heidelberg, Germany; 2 Department of Medical Physics, Deutsches Krebsforschungszentrum, Im Neuenheimer Feld 270, 69120 Heidelberg, Germany; 3 Department of Radiation Oncology, Klinikum rechts der Isar, Technische Universität München, Ismaninger Straße 22, 81675 Munich, Germany; 4 Institute of Innovative Radiotherapy (iRT), Helmholtz Zentrum München, Ingolstädter Landstraße 1, 85764, Oberschleißheim, Germany; 5 National Center for Radiation Oncology (NCRO), Heidelberg Institute for Radiation Oncology (HIRO), Im Neuenheimer Feld 400, 69120 Heidelberg, Germany; 6 Heavy Ion Therapy Center (HIT), Heidelberg University Hospital, Im Neuenheimer Feld 450, 69120 Heidelberg, Germany; 7 Institute for Medical Biometry and Informatics (IMBI), Heidelberg University Hospital, Im Neuenheimer Feld 130.3, 69120 Heidelberg, Germany; 8 Clinical Cooperation Unit Radiation Oncology (E050), German Cancer Research Center (dkfz), Im Neuenheimer Feld 280, 69120 Heidelberg, Germany

**Keywords:** Proton therapy, Carbon ion therapy, Active raster-scanning, Recurrence, Toxicity, Quality of life, Repeated radiotherapy, Radiotolerance, Salvage therapy, Meningioma

## Abstract

**Background:**

With the advance of modern irradiation techniques, the role of radiotherapy (RT) for intracranial meningioma has increased significantly throughout the past years. Despite that tumor’s generally favorable outcome with local control rates of up to 90% after ten years, progression after RT does occur. In those cases, re-irradiation is often difficult due to the limited radiation tolerance of the surrounding tissue. The aim of this analysis is to determine the value of particle therapy with its better dose conformity and higher biological efficacy for re-irradiating recurrent intracranial meningioma. It was performed within the framework of the “clinical research group heavy ion therapy” and funded by the German Research Council (DFG, KFO 214).

**Methods:**

Forty-two patients treated with particle RT (protons (*n* = 8) or carbon ions (*n* = 34)) for recurrent intracranial meningioma were included in this analysis. Location of the primary lesion varied, including skull base (*n* = 31), convexity (*n* = 5) and falx (*n* = 6). 74% of the patients were categorized high-risk according to histology with a WHO grading of II (*n* = 25) or III (n = 6), in the remaining cases histology was either WHO grade I (*n* = 10) or unknown (n = 1). Median follow-up was 49,7 months.

**Results:**

In all patients, re-irradiation could be performed safely without interruptions due to side effects. No grade IV or V toxicities according to CTCAE v4.0 were observed. Particle RT offered good overall local control rates with 71% progression-free survival (PFS) after 12 months, 56,5% after 24 months and a median PFS of 34,3 months (95% CI 11,7–56,9). Histology had a significant impact on PFS yielding a median PFS of 25,7 months (95% CI 5,8–45,5) for high-risk histology (WHO grades II and III) while median PFS was not reached for low-risk tumors (WHO grade I) (*p* = 0,03). Median time to local progression was 15,3 months (Q1-Q3 8,08–34,6). Overall survival (OS) after re-irradiation was 89,6% after 12 months and 71,4% after 24 months with a median OS of 61,0 months (95% CI 34,2–87,7). Again, WHO grading had an effect, as median OS for low-risk patients was not reached whereas for high-risk patients it was 45,5 months (95% CI 35,6–55,3).

**Conclusion:**

Re-irradiation using particle therapy is an effective method for the treatment of recurrent meningiomas. Interdisciplinary decision making is necessary to guarantee best treatment for every patient.

## Background

Intracranial meningiomas are among the most frequent primary brain tumors [1]. Although benign in principle, they can afflict severe damage on sensitive intracranial structures, causing substantial morbidity. Several different approaches to the treatment of meningiomas are established. For safely accessible tumors, neurosurgical resection is the treatment of choice, however, in critical locations, e.g. at the skull base, radiation therapy (RT) has been established as a safe and highly effective treatment modality [2–4].

For asymptomatic low-grade lesions found incidentally, a wait-and-see strategy can be adapted and based on regular clinical and imaging follow-up [5]. Good long-term local control rates of up to 95% progression-free survival (PFS) at five years and 60–80% at 10 years in separate series can be achieved if the lesion is easily accessible for complete resection [6]. However, substantial post-operative morbidity can occur if sensitive vascular or neuronal structures are compromised by the resection, such as is the case with large tumors located at the skull base if complete resection is sought. On the other hand, postoperative RT can complement incomplete resection and achieve satisfactory results at low toxicity rates. It is strongly recommended for WHO grade II/III meningiomas and can be a suitable option for salvage treatment in case of recurrence after neurosurgical resection [6, 7]. Non-surgical treatment options include stereotactic radiosurgery (SRS) and fractionated stereotactic radiotherapy (FSRT), achieving local control rates similar to those of complete surgical resection for tumors located in regions not accessible to surgery [8]. In some cases, where the preservation of adjoining radiosensitive tissue is critical or tumor shapes are more complex, intensity modulated radiotherapy (IMRT) can deliver higher dose conformity than conventional SRS or FSRT, achieving excellent local control rates [9]. Particle therapy, such as proton or carbon ion irradiation, is characterized by distinct physical and biological properties. The reduction of integral dose to adjoining healthy tissue with particle therapy could contribute to the reduction of long-term toxicity and is of special interest where prolonged survival is potentially achievable, as applies to the treatment of meningiomas [6]. Furthermore, the higher biological doses that can be delivered by the use of heavy particles such as carbon ions could improve tumor control for high-risk histologies [10, 11]. To date, sparse clinical data is available on particle therapy for meningiomas. A significant prognostic factor for progression-free survival (PFS) as well as overall survival (OS) lies in the histological characteristics of the tumor, with benign WHO grade I meningiomas yielding significantly longer PFS and OS than atypical meningiomas (WHO grade II) and malignant/anaplastic tumors (WHO grade III) showing the lowest local control rates as well as shortest OS [12].

In cases of tumor progression after initial radiotherapy, treatment options are generally limited. Interdisciplinary treatment decisions are usually obtained. Re-irradiation can be indicated in selected cases, depending on the previous dose distribution, time between primary and re-irradiation, location and especially on the vicinity to organs at risk (OAR).

Re-irradiation is generally performed using high-precision techniques; the characteristics of particle therapy offer excellent sparing of normal tissue outside the defined target volume, thus promising a beneficial risk-benefit-profile. The current analysis was performed to evaluate toxicity as well as local control and survival after re-irradiation with protons and carbon ions for recurrent meningiomas.

## Methods

### Patient characteristics

Between 2009 and 2013, forty-four patients with recurrent intracranial meningiomas after having previously received radiotherapy, were re-irradiated using particle therapy. Two patients were lost to follow-up. Thus, for our analysis we took into account forty-two patients. All patients received re-irradiation at Heidelberg Ion Therapy Center (HIT), employing the raster-scanning technique for active beam delivery developed by Haberer et al. [13]. Thirty-four patients received carbon ion therapy, whereas eight patients received proton therapy. Median patient age at re-irradiation was 54 years (range 18 to 77 years), while age at primary diagnosis ranged from 12 to 64 years with a median of 44 years. Tumor location can be divided into three main categories: convexity, falx and skull base, of which the skull base was most common (*n* = 31). Seventy-four percent of the patients were categorized high-risk according to histology with a WHO grading of II (*n* = 25) or III (*n* = 6); in the remaining cases histology was either WHO grade I (*n* = 10) or unknown (n = 1). Patient characteristics are illustrated in Table 1.

**Table 1:** Patient characteristics

Age at re-irradiation (years)				
	Mean (SD)	53		13,4
	Median (Q1-Q3)	53		47–61
	Median (range)	53		18–77
			*n*=	%
Gender				
	male	17		40,5%
	female	25		59,5%
Histology				
	WHO I	10		23,8%
	WHO II	25		59,5%
	WHO III	6		14,3%
	unknown	1		2,4%
Location				
	skull base	31		73,8%
	falx	6		14,3%
	convexity	5		11,9%
Karnofsky performance score				
	≥ 80%	34		81,0%
	< 80%	8		19,0%
Previous radiotherapy				
	IMRT	16		38,1%
	3DCRT	16		38,1%
	SRS/FSRT	8		19,0%
	radiopeptide	1		2,4%
	carbon ions	1		2,4%
Recurrence				
	infield / field border	38		90,5%
	outfield	4		9,5%
Particle therapy				
	protons	8		19,0%
	carbon ions	34		81,0%

### Previous treatment and recurrence

Previous treatment included a number of different modalities and techniques. All patients, except for two, had surgery at least once at some point during previous treatment; in almost all cases a partial resection was performed, in one case only a biopsy was performed. Intensity modulated radiotherapy (IMRT) (*n* = 16) and conventional 3D-planned RT (3DCRT) (n = 16) were the most commonly employed techniques with a median cumulative dose of 52,9 Gy (12,1–62,4 Gy) for IMRT. Of those patients, only two received less than 50 Gy: One patient dropped out of treatment after 12,1 Gy and one patient received a hypofractionated regimen of 11 × 3,8 Gy. Median cumulative dose for 3DCRT was 54 Gy (50,5–55,8 Gy). Seven patients received stereotactic radiosurgery (SRS) at a median dose of 12,1 Gy (12,0–17,0 Gy) and one patient had received FSRT at a cumulative dose of 58,8 Gy. One patient had previously received a radiopeptide therapy with Y-90 DOTATATE at 4,39 Gbq, corresponding to an approximated local dose of 10 Gy, whereas one patient received two consecutive courses of carbon ion RT due to tumor progression. None of the patients received any kind of systemic therapy.

All tumor recurrences were confirmed by repeated imaging via contrast-enhanced CT or MRI, in 17 cases an additional FET- and/or DOTATOC-PET was performed to help treatment planning. In most cases, infield and field border recurrences were observed (*n* = 38), only four cases showed additional outfield growth.

### Target volume delineation

For treatment planning, an individual head fixation mask that guarantees immobilization during RT and allows for precision dose delivery to a maximum positioning error of 1–2 mm was individually fitted for each patient [14, 15]. Correct patient positioning was verified prior to beam delivery using orthogonal X-rays.

For target volume definition, the treatment planning CT imaging data was matched to a contrast-enhanced MRI to allow for a more precise estimate of microscopic tumor extension. On the T1-weighted sequence, contrasted tumor formations were delineated as gross tumor volume (GTV). Adjoining meningeal enhancement (dural tail) was included into the clinical target volume (CTV) and in complex cases with extensive locoregional spread (e.g. infiltration of bony structures, defects and changed anatomy due to previous surgery), a safety margin of 1 mm (benign histology) or 2–3 mm (malignant histology) was added and adapted at the discretion of the treating physician to include areas of potential microscopic spread. In 17 cases, an additional FET- and/or DOTATOC-PET was performed to further facilitate target volume definition. GTV equaled CTV in 29 (69,0%) of the cases. Median relative increase in CTV size was 76,4% (22,7 ml) in the 13 cases where an additional safety margin was added. An isotropic PTV margin of 3 mm was added in all cases to compensate for positioning and technical insecurities, as is standard procedure for intracranial irradiation at HIT. Details of resulting target volume sizes are illustrated in Table 2.

**Table 2:** Target volume sizes and treatment planning parameters.

	median (ml)	Q1-Q3	mean (ml)	std dev (ml)
GTV	18,1	6,7–82,6	51,3	67,9
CTV	48,9	22,5–93,9	82,3	96,3
PTV	75,1	37,1–126,2	102,9	93,6
			n=	%
Cases with GTV = CTV			29	69,0%
Cases with additional CTV margin			13	31,0%
			median	Q1-Q3
median absolute increase by CTV			22,7 ml	9,3 ml - 43,1 ml
Median relative increase by CTV			76,4%	35% - 269,5%

### Treatment planning

Treatment planning and biological plan optimization were done utilizing the planning software TRiP [16, 17]. Patients received a median cumulative dose of 51 Gy(RBE) (range 15–60 Gy(RBE)) of particle therapy at a median of 19 fractions (range 5–32 fractions). Four of those patients received particle therapy only as a carbon ion boost of 15 Gy(RBE) (*n* = 1) or 18 Gy(RBE) (*n* = 3), applied after 50–52 Gy of photon irradiation. For carbon ion therapy most commonly, a dose per fraction of 3 Gy(RBE) was applied as well as a dose per fraction of 3,3 Gy in one case. For proton therapy smaller doses per fraction such as 1,8 Gy(RBE) or 2,0 Gy(RBE) were used. Treatment was delivered in 6 daily fractions per week.

Generally, where OAR tolerance permitted, a dose upward of 50 Gy(RBE) for WHO I tumors and upward of 54 Gy(RBE) for higher grade tumors was aimed for. Coverage by the prescribed dose was optimized for CTV; focally reduced PTV coverage was accepted to allow for OAR sparing if necessary. Actual dose prescription was decided on a case-by-case basis and was naturally influenced by the dose distribution of previous radiotherapy and remaining radiotolerance. Carbon ions were preferred for re-irradiation for their higher biological effectiveness and the potential benefit in tumors that had progressed after previous radiotherapy. Protons were chosen in selected cases where the moderately hypofractionated approach established for carbon ion therapy with a single dose of 3 Gy(RBE) was not preferable or for resulting in an advantageous dose distribution in individual cases. The approach of delivering a carbon ion boost of 18 Gy(RBE) in addition to 50 Gy of photon radiotherapy was applied for patients treated analogous to the MARCIE-trial, a phase II trial currently being conducted at our institution for atypical meningiomas [18]. Table 3 presents an overview of cumulative doses and fractionation schemes used for different histologies.

**Table 3:** Different fractionation schemes and their absolute frequencies listed by tumor histology. To facilitate comparison between fractionation schemes equivalent doses in 2 Gy fractions (EQD2) for an assumed α/β of 2 have been calculated.

Histology	fractionation	cumulative dose (Gy(RBE))	EQD2 (Gy(RBE))	*n*=	median cumulative EQD2 (Gy(RBE))
WHO I	C12: 15 × 3	45	56,3	2	52,4
	H1: 32 × 1,8	57,6	54,7	2	
	C12: 17 × 3	51	63,8	1	
	H1: 29 × 1,8	52,2	49,6	1	
	C12: 25 × 2	50	50,0	1	
	C12: 16 × 3	48	60,0	1	
	H1: 30 × 1,5	45	39,4	1	
	C12: 12 × 3	36	45,0	1	
WHO II	C12: 17 × 3	51	63,8	10	60,0
	C12 Boost after 50 Gy photon RT: 6 × 3	68	72,5	3	
	C12: 13 × 3	39	48,8	2	
	C12: 15 × 3	45	56,3	2	
	C12: 19 × 3	57	71,3	1	
	H1: 27 × 2	54	54,0	1	
	H1: 30 × 1,8	54	51,3	1	
	H1: 25 × 2	50	50,0	1	
	C12: 15 × 3,3	49,5	65,6	1	
	C12: 20 × 3	60	75,0	1	
	C12: 18 × 3	54	67,5	1	
	H1: 20 × 2	40	40,0	1	
	C12 Boost after 50 Gy photon RT: 5 × 3	65	68,8	1	
WHO III	C12: 17 × 3	51	63,8	3	56,3
	C12: 15 × 3	45	56,3	2	
	C12: 13 × 3	39	48,8	1	

For all patients, documentation of previous radiotherapy including multi-slice dose distribution was obtained and carefully correlated with the current clinical situation. Constraints for OAR, including brain stem and optic pathway, were set in consideration of the previous cumulative dose received and generally based on the recommendations laid out by Emami et al. [19]. TD 5/5 dose limits were disregarded in several cases where a clinical rationale justified that decision (e.g. tumor infiltration or direct proximity). In those cases, the increased risk of treatment-associated toxicity was discussed individually with the patient and a decision was reached with respect to patient preference and clinical necessity. Parts of the optic pathway received maximum doses upward of 50 Gy in a total of 9 cases. An overview of biological doses received by OAR is illustrated in Table 4. To compensate for differences in fractionation, equivalent doses in 2 Gy fractions (EQD2) for an assumed α/β of 2 for normal tissue are displayed.

**Table 4:** Dose statistics (EQD2) for different organs at risk, regarded independently by tumor location: All locations (*n* = 41), only skull base tumors (n = 31) and a subgroup of especially complex cases with tumors adjoining to OAR (*n* = 28)

	median mean dose	IQR	mean mean dose	std dev	median max dose	IQR	mean max dose	std dev
All locations, n = 41								
brain stem	1,1	0,1–3,4	2,5	4,6	21,1	1–37,5	23,1	18,1
optic chiasm	3,5	0–11,7	7,7	10,7	19,6	0,2–35,9	20,4	19,3
left optic nerve	2,9	0–11,3	9,8	15,6	20,3	0,1–43,6	23,9	22,9
right optic nerve	1,7	0,1–9	10,7	18,5	16,1	0,3–44,3	24,0	25,3
Only skull base, n = 31								
brain stem	2,0	0,9–3,9	3,4	5,2	31,7	19,9–40,2	31,1	13,9
optic chiasm	5,5	2,7–16	10,3	11,5	23,7	15–41,2	27,2	17,8
left optic nerve	5,9	2,2–21,2	13,7	17,1	29,6	17,7–48,7	32,9	21,1
right optic nerve	4,3	0,8–35,7	14,9	20,5	25,7	11–54,2	32,1	24,9
Complex cases, n = 28								
brain stem	2,0	0,9–3,8	3,4	5,2	31,7	20,2–38,4	30,3	12,6
optic chiasm	6,0	2,9–16,6	10,9	11,6	24,6	16,1–40,6	28,1	17,2
left optic nerve	6,1	1,8–23,6	14,5	17,4	29,6	18,6–50,1	34,0	20,9
right optic nerve	2,9	0,8–29,4	14,1	20,1	25,7	12,8–52,4	31,8	24,2

### Follow-up

The first follow-up visit was scheduled 6 weeks after therapy completion with subsequent three-monthly visits for the first year. After that, twice-yearly visits were scheduled for an additional period of 2 years, thereafter once a year. Procedure during follow-up consisted of a contrast-enhanced MRI-examination as well as a thorough clinical check-up [20]. Symptoms and toxicities were documented in detail in the patient’s medical record and subsequently entered into a prospective research database maintained at our institution for long-term systematic follow-up of radiooncological patients [21]. Symptoms were classified according to the Common Terminology Criteria for Adverse Events (CTCAE) v. 4.0 [22]. New or worsening symptoms were considered acute and treatment-related toxicities if they occurred within the first 6 months after radiotherapy and late toxicities if they occurred after that. Symptoms were followed up and outcome was judged at last follow-up as either stable/improved or worsened. Toxicities of grades I and II according to CTCAE were classified low-grade. Any de novo symptoms grade III or higher were classified high-grade, as were any pre-existing symptoms worsening by at least two CTCAE grades except if directly attributable to tumor progression.

### Statistical analysis

For descriptive baseline analyses, continuous variables are given as means (SD) and median (quartiles, range where appropriate) and categorical variables as absolute and relative frequencies. Overall survival (OS) was calculated separately from the date of primary diagnosis and from the date of re-irradiation until death or last observation during follow-up (censored data). Progression-free survival (PFS) was determined from the time of the beginning of radiotherapy until tumor progression or to last observation or death if none occurred (censored data). OS and PFS were calculated using the Kaplan-Meier-Method. The median follow-up time was calculated using the reverse Kaplan-Meier method [23]. Survival curves for prognostic factors were compared using a two-sided log-rank test. Since this was a retrospective exploratory data analysis, *p*-values are of descriptive nature. A descriptive p-value of < 0.05 was considered to indicate statistical significance. All statistical analyses were performed using the statistics software *IBM SPSS Statistics Version 22 (New York, USA).* This study was approved by the Ethics Committee of the Medical Faculty of Heidelberg (ref. no.: s-207/2013).

## Results

### Local tumor control and survival

**Fig. 1:**
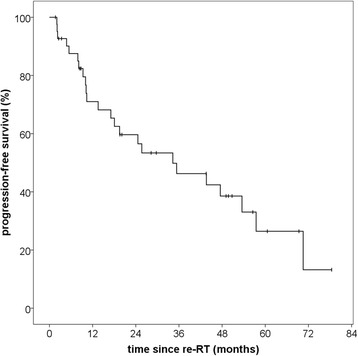
Progression-free survival for patients with recurrent meningioma regardless of histology after re-irradiation with particle therapy

**Fig. 2:**
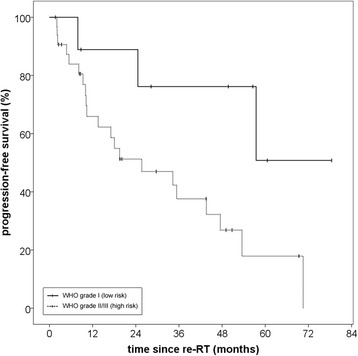
Impact of histology on progression-free survival when classified as low-risk (WHO grade I) and high-risk (grades II and III). The difference between the two groups was significant (*p* = 0,03)

**Fig. 3:**
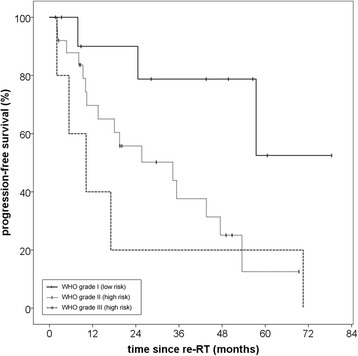
Impact of histology on progression-free survival regarding all WHO grades separately: The difference between grades I and III was significant (*p* = 0.02) but not between grades II and III (p = 0,43)

The reverse Kaplan-Meier estimate for median follow-up was 49,7 (Q1-Q3 28,3–69,4; 95% CI 29,7–60,6) for progression-free survival and 50,2 months (Q1-Q3 23,2–64,6; 95% CI 42,8–56,1) for overall survival. A progression-free survival rate (PFS) of 71,0% after 12 months and 56,5% after 24 months could be achieved. Median PFS for all patients was 34,3 months (Q1-Q3 10,2–70,5; 95% CI 11,7–56,9) (Fig. 1). Histology at primary diagnosis appeared to be an important prognostic factor for progression-free survival as well as overall survival (OS) with a clear distinction between low-risk (WHO grade I histology) and high-risk tumors (WHO grades II and III). Histology-adjusted median PFS showed to be 25,7 months (Q1-Q3 10,0–54,0; 95% CI 5,8–45,5) for high-risk tumors, while for low-risk tumors median PFS was not reached due to the limited number of events. The difference in PFS between low-risk and high-risk tumors was significant (*p* = 0,03) (Fig. 2). Regarding all three WHO grades separately, median PFS for grade II meningiomas was 34,3 months (Q1-Q3 10,0–54,0; 95% CI 6,9–61,7) and for grade III meningiomas 10,2 months (Q1-Q3 5,4–17,1; 95% CI 0–20,4). While patient numbers are limited in this analysis, no significant difference in PFS could be detected between grade II and grade III meningiomas (*p* = 0,43) (Fig. 3).

**Fig. 4:**
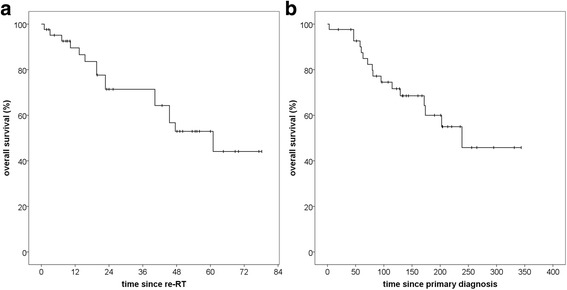
Overall survival for patients with recurrent meningioma regardless of histology after re-irradiation with particle therapy, calculated from date of re-irradiation (**a**) and from date of primary diagnosis (**b**)

**Fig. 5:**
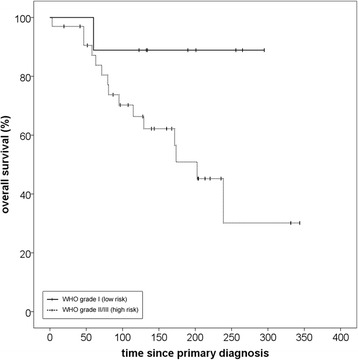
Impact of histology on overall survival when classified as low-risk (WHO grade I) and high-risk (grades II and III). While patient number is limited, statistical significance was not reached (*p* = 0.05)

**Fig. 6:**
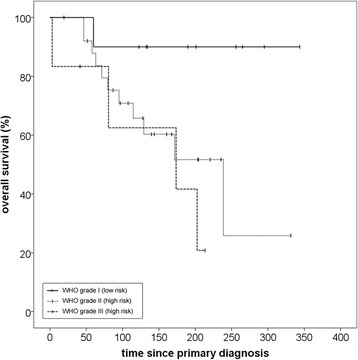
Impact of histology on overall survival regarding all WHO grades separately: The difference between grades I and III was significant (p = 0,01) but not between grades II and III (p = 0,30)

Overall survival (OS) after re-RT was 89,6% after 12 months and 71,4% after 24 months with a median overall survival of 61,0 months (95% CI 34,2–87,7) (Fig. 4a). Calculated from the date of primary diagnosis, median OS was 238,7 months (Fig. 4b). Again, histology at primary diagnosis appeared to be an important prognostic factor, albeit statistical significance was not reached (p = 0,05), possibly due to small sample size. Median OS was not reached in the low-risk group. One death unrelated to meningioma was documented in this group. In the high-risk group median OS was 202,5 months (95% CI 149,3–255,8) (p = 0,05) (Fig. 5). Regarding all three WHO grades separately, median OS was significantly better for grade I, compared to both grades II and III. Median OS was not reached for grade I and was 238,7 months (95% CI 118,8–358,6) for grade II (p = 0,04). For grade III median OS was 173,6 (95% CI 0–367,7) (p = 0,02)). There was no significant difference in OS between grade II and grade III (p = 0,38) (Fig. 6).

Progression after re-radiotherapy in all cases occurred as local progression. Median time to progression was 15,3 months (Q1-Q3 8,08–34,6) with tumor progression occurring within the first 24 months after re-irradiation in 63% of the cases. In most cases, tumor progression occurred in-field (*n* = 10) or at the field border (*n* = 7). Five patients developed out-of-field tumor progression in the form of secondary intracranial meningiomas; of those cases three also developed in-field progression. In two cases, the exact site of tumor progression could not be determined for imaging data was not available at our institution.

### Treatment-related toxicity

**Fig. 7:**
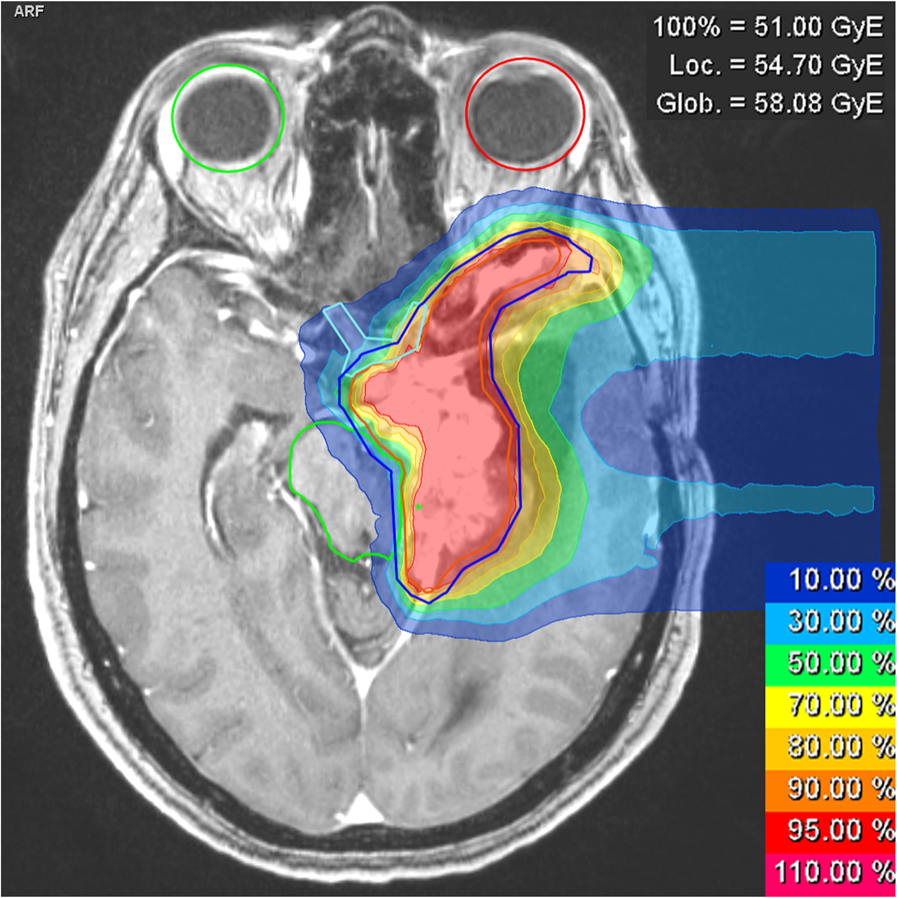
Exemplary treatment plan for re-irradiation of a large recurrent meningioma of the skull base. A re-irradiation dose of 17 × 3 Gy(RBE) carbon ions was applied and a dose of 11 × 3,8 Gy photons had been applied one year earlier in a FSRT-setting. Dose to the directly adjoining optic chiasm could be reduced to 11,0 Gy(RBE) mean (33,3 Gy(RBE) max) and dose to the brain stem to 6,5 Gy(RBE) mean (36,3 Gy(RBE) max). CTV is delineated in red and PTV in blue

All patients were able to complete re-irradiation successfully and no interruptions or abortions of treatment due to acute toxicity were necessary. Exploiting the physical and biological features of particle irradiation, a high dose conformity could be achieved in treatment planning, effectively reducing dose to adjoining OAR with depleted radiotolerance due to previous irradiation (Fig. 7). No grade IV or V toxicities according to CTCAE v4.0 were observed. Acute toxicity was moderate and included mostly focal alopecia, fatigue and moderate skin irritation. In all cases, acute toxicity was regressive within a maximum of one year after completion of therapy. Few cases of late toxicity were observed, including predominantly prolonged fatigue, low grade xerostomia and intermittent headaches or episodes of nausea (Table 5).

**Table 5:** Acute and late treatment-related toxicity

	Acute treatment-related toxicity				Late treatment-related toxicity			
Side effect	low grade (CTCAE I-II)		high grade (CTCAE III or higher)		low grade (CTCAE I-II)		high grade (CTCAE III or higher)	
	*n*=	%	*n*=	%	*n*=	%	*n*=	%
focal alopecia	14	33,3%	0	0,0%	1	2,4%	0	0,0%
fatigue	11	26,2%	0	0,0%	4	9,5%	0	0,0%
skin irritation	10	23,8%	0	0,0%	1	2,4%	0	0,0%
headache	7	16,7%	0	0,0%	2	4,8%	0	0,0%
nausea	7	16,7%	0	0,0%	2	4,8%	0	0,0%
lymphedema	3	7,1%	0	0,0%	0	0,0%	0	0,0%
mucositis	3	7,1%	0	0,0%	0	0,0%	0	0,0%
xerostomia	2	4,8%	0	0,0%	3	7,1%	0	0,0%
facial pain	1	2,4%	0	0,0%	2	4,8%	0	0,0%
radionecrosis	1	2,4%	2	4,8%	0	0,0%	0	0,0%
dysgeusia	1	2,4%	0	0,0%	0	0,0%	0	0,0%

Three cases of radionecrosis were documented: One patient with an atypical meningioma of the left cerebellar tentorium was treated with 51 Gy(RBE) carbon ions after having received 54 Gy adjuvant photon radiotherapy in an overlapping area, three years prior. Radionecrosis was symptomatic with blurred vision and dizziness. In addition to the radionecrosis, the patient developed tumor progression at the same time and was referred to neurosurgical resection. In the second case, the patient had received 60 Gy of adjuvant photon radiotherapy after resection of an anaplastic meningioma of the right sphenoid wing in 2011. He was re-irradiated one year later in 2012 for local progression, receiving 51 Gy(RBE) carbon ions that he tolerated well. He developed a radionecrosis of the right temporal lobe after receiving 40 Gy of another course of photon irradiation in 2013 for a second anaplastic meningioma of the falx that he developed during follow-up. The patient was referred to successful neurosurgical necrosectomy but died 7 months thereafter of tumor progression. Notably he had also been previously irradiated for retinoblastoma in 1969. In the third case, radionecrosis developed after re-irradiation with 51 Gy(RBE) carbon ions and previous 60 Gy of photon irradiation two years prior for an anaplastic meningioma of the parieto-occipital region. Symptoms were mild and did not progress after corticosteroid administration. They consisted of slight worsening of pre-existing epilepsy, blurred vision and headaches.

### Symptom response to treatment

Symptoms and neurological status were ascertained before the beginning of treatment, during treatment as well as during follow-up. Common symptoms prior to RT included pain or neuralgia in the head-and-neck region, neurological symptoms in terms of motor impairment, hypoesthesia or paresthesia, mostly also in the facial or head-and-neck region, hearing impairment, dizziness, seizures and visual impairment. Notably, visual impairment, mostly diplopia, proved to be among the most common symptoms prior to radiotherapy. In 24% of the affected patients, an improvement within a year after re-irradiation could be achieved. During long-term follow-up, a total of four patients reported a worsening of their visual impairment after re-irradiation with particle therapy. Motor impairment improved or stabilized in 31% of the affected patients and sensory impairment in 38%. Overall, few patients reported worsening of their symptoms during follow-up and second to eye-related symptoms predominantly motor function was affected. An overview of the predominant symptoms prior to re-irradiation and their relative development after re-irradiation is presented in Table 6. No secondary malignancies in the brain or head-and-neck area were reported.

**Table 6:** Predominant symptoms prior to re-irradiation and their relative improvement development during follow-up

	Symptoms before particle re-irradiation			Symptoms at last follow-up					Clinical outcome			
Predominant clinical symptoms	low grade (CTCAE I-II)	high grade (CTCAE III or higher)		low grade (CTCAE I-II)		high grade (CTCAE III or higher)			stable or improvement	worsening		
	*n*=	%	*n*=	%	*n*=	%	*n*=	%	*n*=	%	*n*=	%
sensory impairment	17	40,5%	1	2,4%	14	33,3%	1	2,4%	16	38,1%	1	2,4%
motor impairment	12	28,6%	3	7,1%	9	21,4%	1	2,4%	13	31,0%	3	7,1%
visual impairment	11	26,2%	6	14,3%	7	16,7%	9	21,4%	12	28,6%	4	9,5%
cognitive impairment	9	21,4%	0	0,0%	5	11,9%	1	2,4%	10	23,8%	0	0,0%
hearing impairment	7	16,7%	3	7,1%	9	21,4%	3	7,1%	10	23,8%	2	4,8%
headaches	6	14,3%	0	0,0%	7	16,7%	0	0,0%	10	23,8%	2	4,8%
seizures	5	11,9%	0	0,0%	4	9,5%	0	0,0%	6	14,3%	1	2,4%
nausea	4	9,5%	0	0,0%	3	7,1%	1	2,4%	8	19,0%	2	4,8%
dizziness	2	4,8%	1	2,4%	3	7,1%	1	2,4%	4	9,5%	2	4,8%
facial pain	2	4,8%	1	2,4%	3	7,1%	1	2,4%	4	9,5%	2	4,8%
fatigue	1	2,4%	0	0,0%	5	11,9%	0	0,0%	9	21,4%	2	4,8%

## Discussion

The present analysis demonstrates that re-irradiation with particle therapy offers a low toxicity profile; in spite of the reduced doses in re-irradiation, local control is relatively high at 71% after 12 months and survival after re-irradiation is promising.

Recurrences after RT in patients with meningiomas generally represent a difficult clinical situation; previous radiotherapy has often fully exhausted the radiation tolerance of surrounding normal tissue; thus, any additional RT has to be performed using highly advanced RT modalities. Other treatment alternatives include surgery, however, especially in skull base lesions, the risk of neurosurgical intervention can be associated with high rates of treatment-related sequelae [6]. Systemic treatment offers only modest effect: Smaller series on chemotherapeutic substances such as Hydroxyurea and temozolomide offer only limited efficacy, however, can by associated with significant hematological toxicity [24, 25]. Molecularly targeted substances, such as VEGFR and EGFR inhibitors have been applied in individual patients after neuropathological evaluation of marker expression, however, overall results were poor and no larger series or randomized trials are available. Moderate results have been shown in small retrospective series for the angiogenesis inhibitor bevacizumab with a median PFS of 18 months although significant toxicity was reported, with one fifth of the included patients discontinuing therapy due to toxicity [26]. Comparable results were found for treatment with sunitinib, a small-molecule tyrosine kinase inhibitor targeting VEGFR with a reported six-months-PFS rate (PFS-6) of 42%. However, here again one third of the included patients required dose reduction and 22% were removed from the study due to increased toxicity including one fatal CNS hemorrhage [27]. An overview of the limited systemic treatment options for recurrent meningioma has been provided by Kaley and colleagues, who in 2014 reviewed forty-seven different publications on the subject and calculated a weighted average PFS-6 of 29% for WHO grade I meningioma and 26% for WHO grade II/III meningioma respectively [28].

Thus, in cases of meningioma recurrence after RT treatment options are limited, and a second course of RT is discussed frequently when no other alternatives are available. Although high precision photon RT modalities such as SRS/FSRT and IMRT are widely available by now, particle therapy still offers several distinct advantages due to its unique physical characteristics that allow a local dose peak (Bragg Peak) at a variably definable depth level with very little dose deposition up to and beyond that point [29]. Over the past years, several planning studies have shown repeatedly that particle therapy can deliver higher dose conformity, with maximum dose applied to the tumor and reduction of medium and low dose to the surrounding tissue, thus reducing the overall integral dose and effectively sparing OAR [30–32]. This has recently and comprehensively been reviewed by De Ruysscher and colleagues [33]. Particle therapy employing passive methods of beam delivery has been in use at several institutions for some time, however the method of active raster-scanning [13], with which beam delivery is being conducted at HIT is to date unique and has proven advantageous over passive beam delivery in different aspects, since no additional patient-specific hardware is required for the accurate shaping of dose distribution, significantly facilitating and accelerating planning as well as treatment processes [10].

In addition to the abovementioned physical advantages of particle therapy, heavy ions such as carbon offer biological benefits attributed to the increased relative biological effectiveness (RBE) of heavy ion irradiation [16] and decisively affecting treatment planning and effective dose calculation. In vitro experiments have proven the increased cytotoxic effect of carbon ion RT, yielding different values for the RBE depending on factors such as linear energy transfer (LET) value and cell line [34] and showing enhanced cytotoxicity even for relatively radioresistant cells such as pancreatic cancer cells with calculated RBE values of up to 4,5 compared to photon RT [11]. Clinical correlation for this data can be found in studies that have been conducted for several tumor entities such as chordoma, skull base chondrosarcoma as well as adenoid cystic carcinoma, showing improved local control after irradiation with carbon ions compared to photon RT [35–37].

Altogether, the abovementioned aspects prove beneficial in treating a recurrent tumor that is in close vicinity to radiosensitive OAR, especially in a heavily pre-treated situation, as is the case for the patients in this analysis. There have been few studies to date that showed the feasibility and effectiveness of carbon ion RT in the setting of re-irradiation, showing local tumor control of up to 92% at 24 months and 64% at 36 months for different tumor entities of the skull base [20] and only moderate toxicity for recurrent head and neck cancers with different histologies [38].

For the treatment of meningioma, feasibility of particle therapy has been proven in past studies, however the available data focuses mainly on treatment in a primary or adjuvant setting with no prior course of RT, usually including only small groups of patients. Reported survival rates were up to 75% at 5 years and 63% at 7 years for high-risk meningiomas [39] and a more recent analysis employing additional DOTATOC-PET for target volume definition has shown 100% local control (follow-up 2–22 months) for WHO grade I meningiomas [10].

Taking into account those results, there is sparse clinical data available on particle therapy for patients with recurrent meningioma. The abovementioned studies have reported on smaller patient sub-groups receiving helical tomotherapy (*n* = 4) or particle therapy (*n* = 19) as re-irradiation yielding local control rates of up to 67% at 12 months for carbon ion RT [10, 40]. Furthermore, a series on nineteen patients receiving SRS or FSRT as re-irradiation for recurrent meningioma has yielded similar PFS rates and once more proven histology to be the most important prognostic factor for PFS [41]. Limitations of this present study include its retrospective character, limited number of patients, as well as relatively short follow-up. To date, however, there is no other dedicated analysis focusing primarily on the setting of re-irradiation and the use of particle therapy for recurrent meningioma and featuring a comparable cohort size.

## Conclusion

Particle therapy applied as re-irradiation in recurrent meningiomas is a feasible method of achieving good local control at moderate toxicity. Improved dose conformity and thus the reduction of integral dose to OAR potentially leads to substantial clinical benefits. In addition, carbon ions provide an increased relative biological effectiveness, which could be beneficial to tumor control. A longer follow-up and prospective clinical studies on a larger number of patients are necessary to more accurately validate the real value of particle re-irradiation in recurrent meningiomas.

## Abbreviations

CTCAE: Common Terminology Criteria for Adverse Events
FSRT: Fractionated stereotactic radiotherapy
IMRT: Intensity-modulated radiotherapy
OAR: Organ at risk
OS: Overall survival
PFS: Progression-free survival
RBE: Relative biological effectiveness
RT: Radiotherapy
SRS: Stereotactic radiosurgery
WHO: World health organization
